# Redetermnation of lagochiline monohydrate

**DOI:** 10.1107/S1600536810017800

**Published:** 2010-05-19

**Authors:** Aziz Ibragimov, Davron Dolimov, Samat Talipov, Lidiya Izotova, Umardjon Zainutdinov

**Affiliations:** a National University of Uzbekistan, The Faculty of Chemistry, Vuzgorodok 174, Tashkent 100174, Uzbekistan; b Institute of Bioorganic Chemistry, Academy of Sciences of Uzbekistan, H. Abdullaev Str. 83, Tashkent 100125, Uzbekistan

## Abstract

In the title compound, C_20_H_36_O_5_·H_2_O, previously studied by film methods [Vorontsova *et al.* (1975 ▶). *Izvest. USSR Ser. Chem.* **2**, 338–343], the H atoms have been located and the absolute structure (seven stereogenic centres) established. An intra­molecular O—H⋯O hydrogen bond generates an *S*(6) ring. In the crystal, mol­ecules are linked by O—H⋯O hydrogen bonds, forming a three-dimensional network.

## Related literature

For biological and medicinal background to lagochiline [systematic name (6*S*,2*R*)-2,12-bis­(hydroxy­meth­yl)-12-(2-hydroxy­ethyl)-2,6,8-trimethyl­spiro­[bicyclo­[4.4.0]decane-7,5′-oxolane]-3-ol, see: Abramov *et al.* (1958 ▶); Akopov & Ibragimov (1961 ▶); Islamov *et al.* (1990 ▶); Izotova *et al.* (1997 ▶). For the previous structure determination, see: Vorontsova *et al.* (1975 ▶). For ring conformations, see: Evans & Boeyens (1989 ▶).
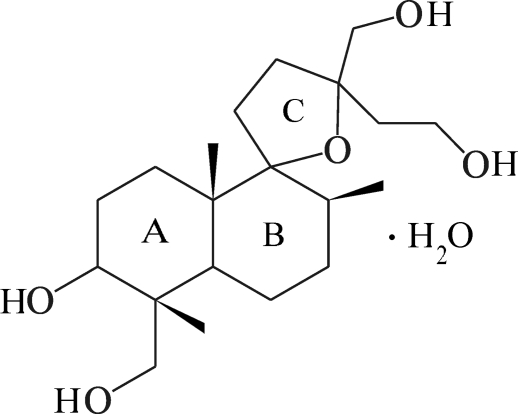


## Experimental

### 

#### Crystal data

C_20_H_36_O_5_·H_2_O
*M* *_r_* = 374.50
Orthorhombic, 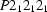

*a* = 7.28495 (14) Å
*b* = 12.5933 (3) Å
*c* = 22.7324 (5) Å
*V* = 2085.51 (7) Å^3^
*Z* = 4
Cu *K*α radiation
μ = 0.70 mm^−1^
*T* = 293 K
0.05 × 0.01 × 0.01 mm

#### Data collection

Oxford Diffraction Xcalibur Ruby diffractometer
Absorption correction: multi-scan (*CrysAlis PRO*; Oxford Diffraction, 2007 ▶) *T*_min_ = 0.899, *T*_max_ = 0.993
8185 measured reflections
4155 independent reflections
3464 reflections with *I* > 2σ(*I*)
*R*_int_ = 0.036
3 standard reflections every 100 reflections  intensity decay: 2.6%

#### Refinement

*R*[*F* ^2^ > 2σ(*F* ^2^)] = 0.042
*wR*(*F* ^2^) = 0.111
*S* = 0.98
4155 reflections
258 parameters
H atoms treated by a mixture of independent and constrained refinement
Δρ_max_ = 0.31 e Å^−3^
Δρ_min_ = −0.22 e Å^−3^
Absolute structure: Flack (1983 ▶), 1674 Friedel pairs
Flack parameter: 0.2 (2)

### 

Data collection: *CrysAlis PRO* (Oxford Diffraction, 2007 ▶); cell refinement: *CrysAlis PRO*; data reduction: *CrysAlis PRO*; program(s) used to solve structure: *SHELXS97* (Sheldrick, 2008 ▶); program(s) used to refine structure: *SHELXL97* (Sheldrick, 2008 ▶); molecular graphics: *XP* (Siemens, 1994 ▶); software used to prepare material for publication: *SHELXL97* and *PLATON* (Spek, 2009 ▶).

## Supplementary Material

Crystal structure: contains datablocks I, global. DOI: 10.1107/S1600536810017800/hb5443sup1.cif

Structure factors: contains datablocks I. DOI: 10.1107/S1600536810017800/hb5443Isup2.hkl

Additional supplementary materials:  crystallographic information; 3D view; checkCIF report

## Figures and Tables

**Table 1:** Hydrogen-bond geometry (Å, °)

*D*—H⋯*A*	*D*—H	H⋯*A*	*D*⋯*A*	*D*—H⋯*A*
O4—H4*O*⋯O5	0.81 (4)	1.87 (4)	2.605 (3)	149 (3)
O5—H5*O*⋯O1*W*	0.77 (5)	1.89 (5)	2.653 (3)	171 (5)
O2—H2*O*⋯O3^i^	0.74 (3)	2.00 (4)	2.732 (2)	173 (3)
O3—H3*O*⋯O2^ii^	0.71 (3)	1.98 (3)	2.684 (2)	177 (3)
O1*W*—H1*W*⋯O3^iii^	0.98 (5)	1.97 (5)	2.916 (4)	161 (4)
O1*W*—H2*W*⋯O4^iv^	0.94 (5)	1.82 (5)	2.722 (3)	160 (5)

Symmetry codes: (i) 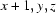; (ii) 
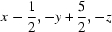
; (iii) 
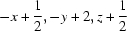
; (iv) 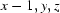.

**Fig. 1.**
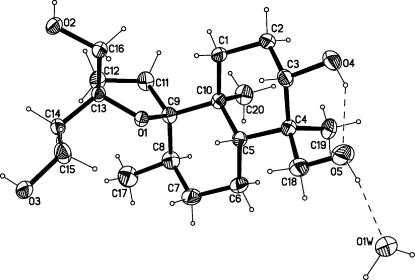
Perspective view of the title compound, showing 30% probability displacement ellipsoids for the non-H atoms. Dashed lines represent hydrogen bonds.

**Fig. 2.**
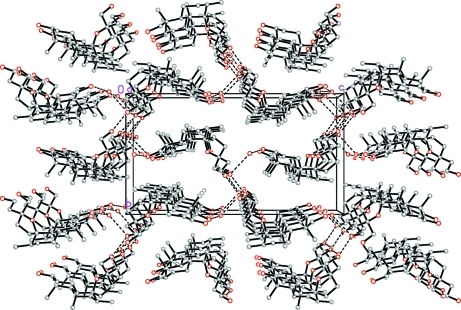
Packing diagram of the title compound (I) viewed down the a axis. H atoms have been ommited for clarity. Hydrogen bonds are shown as dashed lines

## supplementary crystallographic information

### Comment

Lagochiline (I, scheme) is a biologically active diterpenoid isolated from plants of the *Lagochilus kind (*Abramov *et al.*, 1958). It can be used as starting materials for preparing of the important medicinal substances, in particular as high effective hemostatic drug lagochiline (Akopov & Ibragimov,1961) and its synthetic derivative lagodene (Islamov *et al.*, 1990). Lagochiline may be obtained in two crystal forms: as monohydrate at ambient conditions and as anhydrate by crystallization at high temperatures (Izotova *et al.*,1997). The crystal structure of the monohydrate form has been solved 35 years ago (Vorontsova *et al.*, 1975). In this study we report improved structure of the lagochiline monohydrate. Six-membered rings A and B are slightly distorted from the chair form and trans-conjugated, while five-membered ring C is in the half-chair conformation (Evans & Boeyens, 1989).The molecule I has following 7 asymmetric atoms - C3, C4, C5, C8, C9, C10, C13. The value of the Flack parameters 0.2 (2) (Flack, 1983) allows to establish the absolute configuration of the asymmetric centers as: C(3)—S, C(4)—R, C(5)—S, C(8)—R, C(9)—R, C(10)—S, C(13)—S (Spek, 2009). Lagochiline molecule has intramolecular H-bond [H···O 1.87 (4) Å] between O(4)—H and O(5) atoms (Table). Four hydroxyl groups of the molecule (Fig.1), showing protonodonor as well protonoacceptor properties, are involved in the formation of the complicated system of the intermolecular H-bonds in the crystalline state (Table). The water molecule is H-bonded to three molecules of lagochiline: as acceptor (O5—H···O1W) and twice as donors (O1W—H···O4, O1W—H···O3) of protons. In result, three molecules of lagochiline and one water molecule form two-dimensional sheet parallel to the [010]. These sheets are sewed one with another *via *H-bonds O(3)—H···O(2) and O(2)—H···O(3) into three-dimensional network.(Table)(Fig.2)

### Experimental

The extracting of the lagochiline was preformed according to the method in Abramov *et al.* (1958).  Colourless needles of (I) were grown by slow evaporation of a solution in acetone.

### Refinement

H-atoms bonded to carbon were positioned geometrically and refined using a riding model, with U_iso_(H)=1.2 or 1.5 times U_eq_(C). The positions of the hydrogen atoms at the hydroxyl groups of the lagochiline molecule and water have been gained from the difference Fourier map

### Crystal data

**Table:**

C_20_H_36_O_5_·H_2_O	*F*(000) = 824
*M**_r_* = 374.50	*D*_x_ = 1.193 Mg m^−^^3^
Orthorhombic, *P*2_1_2_1_2_1_	Cu *K*α radiation, λ = 1.54184 Å
Hall symbol: P 2ac 2ab	Cell parameters from 1210 reflections
*a* = 7.28495 (14) Å	θ = 3.5–72.7°
*b* = 12.5933 (3) Å	µ = 0.70 mm^−^^1^
*c* = 22.7324 (5) Å	*T* = 293 K
*V* = 2085.51 (7) Å^3^	Needle, colourless
*Z* = 4	0.05 × 0.01 × 0.01 mm

### Data collection

**Table:**

Oxford Diffraction Xcalibur Ruby diffractometer	3464 reflections with *I* > 2σ(*I*)
Radiation source: fine-focus sealed tube	*R*_int_ = 0.036
graphite	θ_max_ = 75.5°, θ_min_ = 3.9°
/ω scans	*h* = −8→5
Absorption correction: multi-scan (*CrysAlis PRO*; Oxford Diffraction, 2007)	*k* = −15→9
*T*_min_ = 0.899, *T*_max_ = 0.993	*l* = −28→27
8185 measured reflections	3 standard reflections every 100 reflections
4155 independent reflections	intensity decay: 2.6%

### Refinement

**Table:**

Refinement on *F*^2^	Hydrogen site location: inferred from neighbouring sites
Least-squares matrix: full	H atoms treated by a mixture of independent and constrained refinement
*R*[*F*^2^ > 2σ(*F*^2^)] = 0.042	*w* = 1/[σ^2^(*F*_o_^2^) + (0.0695*P*)^2^] where *P* = (*F*_o_^2^ + 2*F*_c_^2^)/3
*wR*(*F*^2^) = 0.111	(Δ/σ)_max_ < 0.001
*S* = 0.98	Δρ_max_ = 0.31 e Å^−^^3^
4155 reflections	Δρ_min_ = −0.22 e Å^−^^3^
258 parameters	Extinction correction: *SHELXL97* (Sheldrick, 2008), Fc^*^=kFc[1+0.001xFc^2^λ^3^/sin(2θ)]^-1/4^
0 restraints	Extinction coefficient: 0.0016 (3)
Primary atom site location: structure-invariant direct methods	Absolute structure: Flack (1983), 1674 Friedel pairs
Secondary atom site location: difference Fourier map	Flack parameter: 0.2 (2)

### Special details

**Table:**

Geometry. All esds (except the esd in the dihedral angle between two l.s. planes) are estimated using the full covariance matrix. The cell esds are taken into account individually in the estimation of esds in distances, angles and torsion angles; correlations between esds in cell parameters are only used when they are defined by crystal symmetry. An approximate (isotropic) treatment of cell esds is used for estimating esds involving l.s. planes.
Refinement. Refinement of *F*^2^ against ALL reflections. The weighted *R*-factor wR and goodness of fit *S* are based on *F*^2^, conventional *R*-factors *R* are based on *F*, with *F* set to zero for negative *F*^2^. The threshold expression of *F*^2^ > σ(*F*^2^) is used only for calculating *R*-factors(gt) etc. and is not relevant to the choice of reflections for refinement. *R*-factors based on *F*^2^ are statistically about twice as large as those based on *F*, and *R*- factors based on ALL data will be even larger.

### Fractional atomic coordinates and isotropic or equivalent isotropic displacement parameters (Å^2^)

**Table:**

	*x*	*y*	*z*	*U*_iso_*/*U*_eq_	
O1	0.73266 (16)	0.98222 (10)	0.12261 (5)	0.0312 (3)	
O2	1.0119 (2)	1.17914 (15)	0.04457 (8)	0.0533 (4)	
O3	0.3784 (2)	1.16130 (15)	0.02097 (8)	0.0512 (4)	
O4	0.8884 (3)	1.03744 (17)	0.37419 (7)	0.0586 (5)	
O5	0.5340 (3)	1.0208 (2)	0.38640 (8)	0.0722 (6)	
C1	0.9928 (3)	0.96481 (16)	0.21908 (8)	0.0373 (4)	
H1A	1.1088	0.9470	0.2006	0.045*	
H1B	0.9504	1.0311	0.2022	0.045*	
C2	1.0239 (3)	0.98044 (17)	0.28469 (8)	0.0402 (4)	
H2B	1.0692	0.9150	0.3019	0.048*	
H2C	1.1154	1.0353	0.2909	0.048*	
C3	0.8467 (3)	1.01203 (16)	0.31406 (8)	0.0389 (4)	
H3B	0.8025	1.0769	0.2950	0.047*	
C4	0.6962 (3)	0.92706 (16)	0.30779 (8)	0.0387 (4)	
C6	0.5214 (3)	0.82102 (19)	0.22804 (10)	0.0495 (5)	
H5B	0.5608	0.7516	0.2415	0.059*	
H5C	0.4112	0.8402	0.2495	0.059*	
C7	0.4793 (3)	0.81639 (19)	0.16225 (10)	0.0525 (5)	
H6A	0.3889	0.7613	0.1551	0.063*	
H6B	0.4264	0.8835	0.1500	0.063*	
C8	0.6494 (3)	0.79387 (16)	0.12516 (10)	0.0473 (5)	
H7A	0.6976	0.7249	0.1378	0.057*	
C9	0.8019 (3)	0.87750 (15)	0.13720 (8)	0.0355 (4)	
C10	0.8523 (3)	0.87711 (14)	0.20524 (8)	0.0336 (4)	
C5	0.6724 (3)	0.90276 (14)	0.24047 (8)	0.0338 (4)	
H10A	0.6268	0.9692	0.2234	0.041*	
C11	0.9684 (3)	0.86188 (17)	0.09623 (9)	0.0451 (5)	
H11A	1.0817	0.8789	0.1166	0.054*	
H11B	0.9748	0.7891	0.0824	0.054*	
C12	0.9369 (3)	0.93826 (18)	0.04478 (9)	0.0453 (5)	
H12A	0.8718	0.9033	0.0130	0.054*	
H12B	1.0524	0.9656	0.0299	0.054*	
C13	0.8204 (3)	1.02751 (15)	0.07156 (7)	0.0336 (4)	
C14	0.6692 (3)	1.06778 (16)	0.03011 (8)	0.0369 (4)	
H14A	0.7257	1.1085	−0.0013	0.044*	
H14B	0.6087	1.0071	0.0123	0.044*	
C15	0.5266 (3)	1.1358 (2)	0.05985 (9)	0.0508 (5)	
H15A	0.4788	1.0985	0.0939	0.061*	
H15B	0.5835	1.2010	0.0735	0.061*	
C16	0.9416 (3)	1.11968 (17)	0.09280 (9)	0.0403 (4)	
H16A	0.8700	1.1660	0.1180	0.048*	
H16B	1.0428	1.0917	0.1158	0.048*	
C17	0.5903 (5)	0.7817 (2)	0.06070 (12)	0.0669 (7)	
H17A	0.4957	0.7289	0.0579	0.100*	
H17B	0.6938	0.7602	0.0374	0.100*	
H17C	0.5444	0.8483	0.0464	0.100*	
C18	0.5140 (3)	0.9768 (2)	0.32897 (10)	0.0543 (5)	
H18A	0.4766	1.0320	0.3018	0.065*	
H18B	0.4190	0.9228	0.3295	0.065*	
C19	0.7368 (4)	0.8292 (2)	0.34618 (10)	0.0564 (6)	
H19A	0.7492	0.8508	0.3865	0.085*	
H19B	0.8488	0.7965	0.3333	0.085*	
H19C	0.6378	0.7793	0.3427	0.085*	
C20	0.9383 (3)	0.76842 (17)	0.22045 (11)	0.0516 (6)	
H20A	0.9698	0.7668	0.2614	0.077*	
H20B	1.0470	0.7580	0.1973	0.077*	
H20C	0.8517	0.7130	0.2121	0.077*	
H2O	1.112 (5)	1.176 (2)	0.0410 (13)	0.052 (8)*	
H3O	0.415 (4)	1.202 (2)	0.0029 (13)	0.044 (7)*	
H4O	0.789 (5)	1.038 (3)	0.3905 (14)	0.065 (9)*	
H5O	0.443 (7)	1.011 (4)	0.4031 (18)	0.103 (15)*	
O1W	0.2045 (3)	1.0041 (3)	0.43526 (9)	0.1024 (11)	
H1W	0.195 (7)	0.939 (4)	0.459 (2)	0.123*	
H2W	0.099 (8)	0.999 (4)	0.412 (2)	0.123*	

### Atomic displacement parameters (Å^2^)

**Table:**

	*U*^11^	*U*^22^	*U*^33^	*U*^12^	*U*^13^	*U*^23^
O1	0.0317 (6)	0.0317 (6)	0.0301 (5)	0.0050 (5)	0.0028 (5)	0.0027 (5)
O2	0.0319 (8)	0.0696 (10)	0.0584 (9)	−0.0018 (7)	0.0029 (7)	0.0311 (8)
O3	0.0348 (7)	0.0619 (10)	0.0569 (9)	0.0052 (7)	0.0039 (7)	0.0269 (8)
O4	0.0480 (9)	0.0895 (13)	0.0381 (8)	−0.0059 (8)	−0.0057 (7)	−0.0100 (8)
O5	0.0485 (10)	0.1158 (17)	0.0522 (9)	−0.0073 (11)	0.0150 (8)	−0.0232 (11)
C1	0.0300 (8)	0.0431 (9)	0.0390 (9)	−0.0009 (8)	0.0012 (8)	0.0070 (8)
C2	0.0329 (9)	0.0469 (10)	0.0407 (9)	−0.0035 (8)	−0.0050 (8)	0.0078 (8)
C3	0.0391 (10)	0.0444 (10)	0.0332 (8)	0.0022 (8)	−0.0061 (7)	0.0019 (7)
C4	0.0351 (9)	0.0455 (10)	0.0354 (9)	−0.0002 (8)	0.0015 (8)	0.0064 (8)
C6	0.0455 (11)	0.0516 (11)	0.0515 (11)	−0.0167 (10)	0.0020 (10)	0.0032 (10)
C7	0.0499 (13)	0.0519 (11)	0.0557 (13)	−0.0209 (10)	−0.0035 (11)	−0.0052 (10)
C8	0.0576 (13)	0.0356 (10)	0.0486 (11)	−0.0041 (9)	−0.0036 (10)	−0.0061 (8)
C9	0.0377 (9)	0.0311 (8)	0.0378 (9)	0.0071 (7)	−0.0005 (7)	0.0001 (7)
C10	0.0355 (9)	0.0282 (8)	0.0372 (9)	0.0053 (7)	0.0011 (7)	0.0048 (7)
C5	0.0337 (9)	0.0324 (8)	0.0352 (8)	−0.0015 (7)	−0.0021 (7)	0.0068 (7)
C11	0.0489 (12)	0.0437 (10)	0.0426 (10)	0.0164 (9)	0.0054 (9)	−0.0039 (8)
C12	0.0468 (11)	0.0524 (11)	0.0368 (9)	0.0135 (9)	0.0084 (8)	−0.0008 (9)
C13	0.0329 (8)	0.0395 (9)	0.0284 (8)	0.0046 (8)	0.0030 (7)	0.0032 (7)
C14	0.0365 (10)	0.0444 (9)	0.0297 (8)	0.0017 (8)	−0.0011 (7)	0.0026 (7)
C15	0.0469 (11)	0.0659 (13)	0.0396 (10)	0.0200 (11)	0.0006 (9)	0.0080 (10)
C16	0.0348 (10)	0.0479 (10)	0.0382 (9)	−0.0012 (8)	0.0010 (7)	0.0109 (8)
C17	0.0856 (19)	0.0593 (14)	0.0558 (14)	−0.0175 (14)	−0.0086 (13)	−0.0154 (12)
C18	0.0393 (11)	0.0784 (15)	0.0453 (10)	−0.0003 (11)	0.0036 (9)	−0.0044 (11)
C19	0.0661 (15)	0.0584 (13)	0.0447 (11)	−0.0082 (12)	0.0003 (11)	0.0210 (11)
C20	0.0593 (14)	0.0355 (10)	0.0600 (13)	0.0172 (9)	−0.0007 (11)	0.0073 (9)
O1W	0.0453 (10)	0.200 (3)	0.0617 (12)	−0.0183 (15)	−0.0003 (9)	0.0361 (17)

### Geometric parameters (Å, °)

**Table:**

O1—C13	1.442 (2)	C9—C10	1.590 (3)
O1—C9	1.450 (2)	C10—C20	1.545 (2)
O2—C16	1.423 (2)	C10—C5	1.569 (3)
O2—H2O	0.74 (3)	C5—H10A	0.9800
O3—C15	1.432 (3)	C11—C12	1.532 (3)
O3—H3O	0.71 (3)	C11—H11A	0.9700
O4—C3	1.436 (2)	C11—H11B	0.9700
O4—H4O	0.81 (4)	C12—C13	1.534 (3)
O5—C18	1.426 (3)	C12—H12A	0.9700
O5—H5O	0.77 (5)	C12—H12B	0.9700
C1—C2	1.521 (3)	C13—C14	1.536 (3)
C1—C10	1.538 (3)	C13—C16	1.536 (3)
C1—H1A	0.9700	C14—C15	1.507 (3)
C1—H1B	0.9700	C14—H14A	0.9700
C2—C3	1.507 (3)	C14—H14B	0.9700
C2—H2B	0.9700	C15—H15A	0.9700
C2—H2C	0.9700	C15—H15B	0.9700
C3—C4	1.539 (3)	C16—H16A	0.9700
C3—H3B	0.9800	C16—H16B	0.9700
C4—C19	1.539 (3)	C17—H17A	0.9600
C4—C18	1.544 (3)	C17—H17B	0.9600
C4—C5	1.570 (3)	C17—H17C	0.9600
C6—C7	1.528 (3)	C18—H18A	0.9700
C6—C5	1.533 (3)	C18—H18B	0.9700
C6—H5B	0.9700	C19—H19A	0.9600
C6—H5C	0.9700	C19—H19B	0.9600
C7—C8	1.526 (3)	C19—H19C	0.9600
C7—H6A	0.9700	C20—H20A	0.9600
C7—H6B	0.9700	C20—H20B	0.9600
C8—C17	1.535 (3)	C20—H20C	0.9600
C8—C9	1.555 (3)	O1W—H1W	0.98 (5)
C8—H7A	0.9800	O1W—H2W	0.94 (5)
C9—C11	1.542 (3)		
			
C13—O1—C9	112.92 (13)	C10—C5—H10A	104.9
C16—O2—H2O	114 (2)	C4—C5—H10A	104.9
C15—O3—H3O	103 (2)	C12—C11—C9	105.25 (16)
C3—O4—H4O	104 (2)	C12—C11—H11A	110.7
C18—O5—H5O	108 (3)	C9—C11—H11A	110.7
C2—C1—C10	113.10 (15)	C12—C11—H11B	110.7
C2—C1—H1A	109.0	C9—C11—H11B	110.7
C10—C1—H1A	109.0	H11A—C11—H11B	108.8
C2—C1—H1B	109.0	C11—C12—C13	103.90 (15)
C10—C1—H1B	109.0	C11—C12—H12A	111.0
H1A—C1—H1B	107.8	C13—C12—H12A	111.0
C3—C2—C1	109.95 (15)	C11—C12—H12B	111.0
C3—C2—H2B	109.7	C13—C12—H12B	111.0
C1—C2—H2B	109.7	H12A—C12—H12B	109.0
C3—C2—H2C	109.7	O1—C13—C12	105.94 (15)
C1—C2—H2C	109.7	O1—C13—C14	107.83 (14)
H2B—C2—H2C	108.2	C12—C13—C14	113.27 (16)
O4—C3—C2	107.42 (16)	O1—C13—C16	107.50 (14)
O4—C3—C4	113.20 (17)	C12—C13—C16	111.15 (17)
C2—C3—C4	112.69 (17)	C14—C13—C16	110.82 (16)
O4—C3—H3B	107.8	C15—C14—C13	114.03 (15)
C2—C3—H3B	107.8	C15—C14—H14A	108.7
C4—C3—H3B	107.8	C13—C14—H14A	108.7
C3—C4—C19	111.55 (18)	C15—C14—H14B	108.7
C3—C4—C18	107.55 (18)	C13—C14—H14B	108.7
C19—C4—C18	108.23 (19)	H14A—C14—H14B	107.6
C3—C4—C5	107.74 (15)	O3—C15—C14	111.75 (18)
C19—C4—C5	114.70 (18)	O3—C15—H15A	109.3
C18—C4—C5	106.74 (16)	C14—C15—H15A	109.3
C7—C6—C5	110.50 (17)	O3—C15—H15B	109.3
C7—C6—H5B	109.5	C14—C15—H15B	109.3
C5—C6—H5B	109.5	H15A—C15—H15B	107.9
C7—C6—H5C	109.5	O2—C16—C13	111.24 (16)
C5—C6—H5C	109.5	O2—C16—H16A	109.4
H5B—C6—H5C	108.1	C13—C16—H16A	109.4
C8—C7—C6	112.6 (2)	O2—C16—H16B	109.4
C8—C7—H6A	109.1	C13—C16—H16B	109.4
C6—C7—H6A	109.1	H16A—C16—H16B	108.0
C8—C7—H6B	109.1	C8—C17—H17A	109.5
C6—C7—H6B	109.1	C8—C17—H17B	109.5
H6A—C7—H6B	107.8	H17A—C17—H17B	109.5
C7—C8—C17	108.6 (2)	C8—C17—H17C	109.5
C7—C8—C9	110.92 (16)	H17A—C17—H17C	109.5
C17—C8—C9	115.9 (2)	H17B—C17—H17C	109.5
C7—C8—H7A	107.0	O5—C18—C4	110.81 (19)
C17—C8—H7A	107.0	O5—C18—H18A	109.5
C9—C8—H7A	107.0	C4—C18—H18A	109.5
O1—C9—C11	104.56 (15)	O5—C18—H18B	109.5
O1—C9—C8	109.09 (15)	C4—C18—H18B	109.5
C11—C9—C8	111.67 (17)	H18A—C18—H18B	108.1
O1—C9—C10	107.80 (14)	C4—C19—H19A	109.5
C11—C9—C10	113.92 (16)	C4—C19—H19B	109.5
C8—C9—C10	109.53 (16)	H19A—C19—H19B	109.5
C1—C10—C20	108.68 (17)	C4—C19—H19C	109.5
C1—C10—C5	107.68 (15)	H19A—C19—H19C	109.5
C20—C10—C5	114.02 (16)	H19B—C19—H19C	109.5
C1—C10—C9	110.52 (14)	C10—C20—H20A	109.5
C20—C10—C9	108.31 (16)	C10—C20—H20B	109.5
C5—C10—C9	107.63 (14)	H20A—C20—H20B	109.5
C6—C5—C10	111.53 (16)	C10—C20—H20C	109.5
C6—C5—C4	112.94 (16)	H20A—C20—H20C	109.5
C10—C5—C4	116.43 (15)	H20B—C20—H20C	109.5
C6—C5—H10A	104.9	H1W—O1W—H2W	101 (4)
			
C10—C1—C2—C3	−60.6 (2)	C7—C6—C5—C4	169.13 (19)
C1—C2—C3—O4	−173.49 (17)	C1—C10—C5—C6	178.34 (16)
C1—C2—C3—C4	61.1 (2)	C20—C10—C5—C6	−61.0 (2)
O4—C3—C4—C19	−50.2 (2)	C9—C10—C5—C6	59.18 (19)
C2—C3—C4—C19	71.9 (2)	C1—C10—C5—C4	−50.0 (2)
O4—C3—C4—C18	68.3 (2)	C20—C10—C5—C4	70.6 (2)
C2—C3—C4—C18	−169.56 (16)	C9—C10—C5—C4	−169.21 (15)
O4—C3—C4—C5	−176.98 (17)	C3—C4—C5—C6	−178.34 (17)
C2—C3—C4—C5	−54.8 (2)	C19—C4—C5—C6	56.8 (2)
C5—C6—C7—C8	55.4 (3)	C18—C4—C5—C6	−63.1 (2)
C6—C7—C8—C17	175.38 (19)	C3—C4—C5—C10	50.7 (2)
C6—C7—C8—C9	−56.2 (2)	C19—C4—C5—C10	−74.2 (2)
C13—O1—C9—C11	8.33 (19)	C18—C4—C5—C10	165.97 (17)
C13—O1—C9—C8	−111.25 (16)	O1—C9—C11—C12	−22.3 (2)
C13—O1—C9—C10	129.90 (15)	C8—C9—C11—C12	95.5 (2)
C7—C8—C9—O1	−60.0 (2)	C10—C9—C11—C12	−139.73 (17)
C17—C8—C9—O1	64.4 (2)	C9—C11—C12—C13	27.4 (2)
C7—C8—C9—C11	−175.03 (18)	C9—O1—C13—C12	9.1 (2)
C17—C8—C9—C11	−50.7 (3)	C9—O1—C13—C14	130.62 (16)
C7—C8—C9—C10	57.8 (2)	C9—O1—C13—C16	−109.86 (16)
C17—C8—C9—C10	−177.9 (2)	C11—C12—C13—O1	−22.6 (2)
C2—C1—C10—C20	−70.5 (2)	C11—C12—C13—C14	−140.56 (18)
C2—C1—C10—C5	53.5 (2)	C11—C12—C13—C16	93.9 (2)
C2—C1—C10—C9	170.76 (15)	O1—C13—C14—C15	49.4 (2)
O1—C9—C10—C1	−57.31 (19)	C12—C13—C14—C15	166.26 (19)
C11—C9—C10—C1	58.2 (2)	C16—C13—C14—C15	−68.0 (2)
C8—C9—C10—C1	−175.89 (16)	C13—C14—C15—O3	−173.44 (18)
O1—C9—C10—C20	−176.26 (16)	O1—C13—C16—O2	−171.20 (15)
C11—C9—C10—C20	−60.7 (2)	C12—C13—C16—O2	73.3 (2)
C8—C9—C10—C20	65.2 (2)	C14—C13—C16—O2	−53.6 (2)
O1—C9—C10—C5	60.03 (18)	C3—C4—C18—O5	−53.1 (3)
C11—C9—C10—C5	175.57 (16)	C19—C4—C18—O5	67.6 (3)
C8—C9—C10—C5	−58.55 (18)	C5—C4—C18—O5	−168.5 (2)
C7—C6—C5—C10	−57.5 (2)		

### Hydrogen-bond geometry (Å, °)

**Table:**

*D*—H···*A*	*D*—H	H···*A*	*D*···*A*	*D*—H···*A*
O4—H4O···O5	0.81 (4)	1.87 (4)	2.605 (3)	149 (3)
O5—H5O···O1W	0.77 (5)	1.89 (5)	2.653 (3)	171 (5)
O2—H2O···O3^i^	0.74 (3)	2.00 (4)	2.732 (2)	173 (3)
O3—H3O···O2^ii^	0.71 (3)	1.98 (3)	2.684 (2)	177 (3)
O1W—H1W···O3^iii^	0.98 (5)	1.97 (5)	2.916 (4)	161 (4)
O1W—H2W···O4^iv^	0.94 (5)	1.82 (5)	2.722 (3)	160 (5)

Symmetry codes: (i) *x*+1, *y*, *z*; (ii) *x*−1/2, −*y*+5/2, −*z*; (iii) −*x*+1/2, −*y*+2, *z*+1/2; (iv) *x*−1, *y*, *z*.

## References

[bb1] Abramov, M. M., Japarova, S. A. & Ikramov, M. I. (1958). *Uzb. Biol. Zh.* **6**, 55–60

[bb2] Akopov, I. E. & Ibragimov, I. I. (1961). *Pharmacol. Toxicol.* **6**, 39–40.

[bb3] Evans, D. G. & Boeyens, J. C. A. (1989). *Acta Cryst.* B**45**, 581–590.

[bb4] Flack, H. D. (1983). *Acta Cryst.* A**39**, 876–881.

[bb5] Islamov, R., Zainutdinov, U. N., Aslanov, Kh. A., Sadykov, A. S., Danil’chuk, D. N., Yankovskiy, B. A. & Zacharov, V. P. (1990). USSR Patent AS 1293990.

[bb6] Izotova, L. Yu., Beketov, K. M., Talipov, S. A. & Ibragimov, B. T. (1997). *Pol. J. Chem.* **71**, 1037–1044.

[bb7] Oxford Diffraction (2007). *CrysAlis PRO.* Oxford Diffraction Ltd, Abingdon, Oxfordshire, England.

[bb8] Sheldrick, G. M. (2008). *Acta Cryst.* A**64**, 112–122. 10.1107/S0108767307043930 18156677

[bb9] Siemens (1994). *XP.* Siemens Analytical X-ray Instruments Inc., Madison, Wisconsin, USA.

[bb10] Spek, A. L. (2009). *Acta Cryst.* D**65**, 148–155. 10.1107/S090744490804362X PMC2631630 19171970

[bb11] Vorontsova, L. G., Tchijov, O. S., Tarnopolsky, B. L. & Andrianov, V. I. (1975). *Izv. USSR Ser. Chem* **2**, 338–343.

